# Bone Marrow and Karyotype Findings of Patients with Pancytopenia in Southern Iran

**Published:** 2014-07

**Authors:** Akbar Safaei, Mansoureh Shokripour, Navid Omidifar

**Affiliations:** Department of Pathology, School of Medicine, Shiraz University of Medical Sciences, Shiraz, Iran

**Keywords:** Pancytopenia, Myelodysplastic syndrome, Megaloblastic anemia, Cytogenetics, Karyotype

## Abstract

**Background: **Pancytopenia is a manifestation of a wide range of disorders. The main prognostic factor for predicting outcome and response to treatment is based on the underlying cause. To detect the root cause of this problem, depending on other accompanied signs or symptoms, the need for bone marrow examination and other advanced work ups is different at least at the practical level. This study focuses on the karyotype abnormality and to demonstrate the ability of this complimentary study in diagnosis and prognosis of such patients.

**Methods:** In this cross sectional study, bone marrow aspiration samples of all patients with Pancytopenia underwent cytogenetic investigation on bone marrow aspiration. Gathered data were analyzed by SPSS software.

**Results:** Among the 100 eligible patients, 67% revealed hypercellular, 19% had hypocellular and 13% had normocellular marrow. Most common causes of pancytopenia were myelodysplastic syndrome (MDS) (33%), MDS vs. megaloblastic anemia (23%) and acute leukemia (18%). Thirty one patients had karyotype abnormality in which majority (13 patients) were diagnosed as MDS followed by 11 patients with acute leukemia.

**Conclusion: **Beside bone marrow examination, there is a need for more supplementary studies like karyotyping to detect the exact cause of pancytopenia. It is concluded that cytogenetic study on bone marrow aspiration can be a complementary test in diagnosis of pancytopenic patients. However, there are also cases where diagnosis even with implementing bone marrow examination and cytogenetic analysis is not possible. Such patients require more clinical follow-up and investigation.

## Introduction

Pancytopenia is a medical condition in which there is a reduction in the number of red and white blood cells, as well as platelets. Pancytopenia is applicable when two parameters from the full blood count are low.^1^^,^^2^ This phenomenon  is defined as the  presence of three findings that may be the end result of different conditions affecting the bone marrow.^3^^,^^4^ The severity of pancytopenia and the underlying etiology settle on the management and prognosis.^5^^,^^6^ The exact identification of the cause will aid in implementing an appropriate therapy. Pathologic causes of pancytopenia can produce hypocellular marrow due to the decrease in hematopoietic cell production or it can produce normocellular or even hypercellular marrow. Such diverse pathologic causes can be present in different diseases such as aplastic anemia (AA), megaloblastic anemia (MA), myelodysplastic syndromes (MDS), acute leukemia, hairy cell leukemia, myelofibrosis with myeloid metaplasia (MMM), lymphomatous or metastatic involvement of bone marrow, leishmaniasis, hemolytic diseases such as systemic lupus erythematous (SLE), hypersplenism and many other diseases.^4^

For detecting the underlying cause of this problem, depending on the other accompanied signs or symptoms, the need for bone marrow examination is mostly needed at least in practical level.^7^^,^^8^ But in some patients, more complicated and advanced tests are required to find the underlying cause  Having said that, even bone marrow examination is not needed in some suspected cases of MA and the diagnosis is made by measuring the  serum level of folat or B12 or even with a simple trial therapy.^9^

The present study focuses on determining the frequency of cytogenetic abnormality in bone marrow specimen of patients with pancytopenia. 

## Materials and Methods

*Patient Selection and Data Gathering*

This cross sectional study was carried out during 2011-2012 and the sample size was calculated according to formula; 

*n*=(p^ 2 ^_*_q^ 2 ^)/d^ 2 ^=100, α=0.05 β=0.8, d=0.025, p=q=0.05

Consecutive bone marrow aspirations of the pancytopenic patients (performed at/referred to hematopathology ward of Faghihi Hospital, affiliated to Shiraz University of Medical Sciences, Shiraz, Iran) were selected. Heparinized bone marrow aspiration samples were sent to a molecular pathology and cytogenetic lab for performing cytogenetic study. All candid patients had pancytopenia based on their peripheral blood criteria. Pancytopenia which was induced by previous hematologic abnormalities (e.g. leukemia on chemotherapy or any other therapy for cancer) was excluded by taking the history and reading medical records of the patients. Inclusion criteria for pancytopenia consisted of; 1-Hemoglobin, <12 g/dL, 2-Total leukocyte count (TLC), <4000 /µL, 3-Platelet count and <100,000/µL. Bone marrow examination had been done on all patients under aseptic precaution. Examination of the peripheral blood (PBS) and bone marrow aspiration and biopsy (BMA/B) was carried out by an expert pathologist. 

*Cytogenetic Study Was Undertaken as Described Below*

**Culture: **Samples were 2cc heparinized bone marrow aspiration. Based on the WBC count, an appropriate amount of a sample was added to 10cc RPMI culture medium. About 1*10^6^ cells per cc were added to 10cc RPMI. Cultured sample were incubated in 37°C for 30 hours by respectively adding Uridine, 5-ﬂuordeoxyuridine, thymidine and 5-bromodeoxyuridine materials in sterile conditions.

Harvest: Initially 70 µL of 10 µg/ml vial of colcemid was added to the sample and incubated in 37°C for 20 min. After 10min centrifuge at 1500 rpm, 10 cc kcl 0.075 M were added to precipitate material and were incubated in 37°C for 10min. 1cc fixative solution was gradually added to the precipitated sample for 5min in room temperature. After 10min centrifuge at 1500 rpm, 10 cc fixative was added to precipitate. The previous stage was repeated twice and centrifuged again to clear the precipitate. 

**Spreading:** 2-3 drops of the prepared precipitate with appropriate concentration were poured on a wet and clean microscopic glass slide. This would mechanically break the cell membranes and leave the chromosomes separated slightly from each other but in a discrete region occupied by a single cell. Five slides were prepared for each case and were put in a water bath 60°C for 1 h then at room temperature for 24 h.

**Banding: **The slides were stained with Giemsa staining in a special material in systematic order with determinant time according to the standard protocol of such staining. Mild trypsinization of the chromosomes before staining obviously weakens the DNA–protein interactions, giving a defined pattern of alternating light and dark regions after the stain is done (G-banding). Then the slides were visualized under microscope.

Briefly about 1*10^ 6 ^cells per milliliter were added to 10 milliliter RPMI 1640 and cultured samples were incubated at 37°C for 30 hours by respectively adding Uridine and 5-ﬂuordeoxyuridine (after 4 h), thymidine (after 22 h) and 5-bromodeoxyuridine (after 28 h). The samples were treated with 70 µL of 10µg/ml vial of colcemid for 10ml of sample and then metaphase chromosomes were spread and stained by using standard G-banding technique. To perform a cytogenetic (karyotype) analysis, accurate identification of each chromosome and determination of chromosome abnormalities is essential. The first step is to count the number of chromosomes in each cell.

After microscopic evaluation of slides, at least 15 representative metaphase cells were captured and re-evaluated by the computer software (Genetix company-USA). The karyograms were prepared by separating and sorting the chromosomes according to their bandings. The homologous pairs based on special banding structure were recognized and arrayed from large to small with special placement of the pair of sex chromosomes after all other chromosomes. The second step was the determination of structural abnormalities of chromosomes if present. When mosaicism was suspected, at least 30 metaphases were examined. Classification of abnormalities was according to the international chromosome nomenclature (ISCN2009).^10^ Gathered data were meticulously entered into the SPSS 9.1 software and frequency tables were obtained. Analysis was carried out in appropriate circumstances as mentioned in the “Results” section.

## Results

One hundred cases that matched the inclusion criteria of this study were selected with a minimum and maximum age of 1 and 92 years, respectively. The mean age was about 44 years consisting of 63% men and 37 % women. 

Splenomegaly and hepatomegaly were detected in 17% and 4% of the patients respectively. Peripheral blood smear (PBS) findings were shift to the left (13%), blast cells (10%), nucleated RBCs (9%), hypersegmented neutrophils (4%), hyposegmented neutrophils (1%) and giant platelets (1%). It is worth noting that among patients with blasts in their peripheral blood, 8 patients were finally diagnosed as acute leukemia and the rest were placed in MDS category. RBC morphologies in PBS were; normocytic (48%), macrocytic (30%) and microcytic (22%). Bone marrow smears showed dyshematopoiesis in 50% of the patients of which megalodyserythropoiesis was the most frequent (25%). Megaloblastic change without dyserythropoiesis was seen in 8% of the patients. Erythroid hyperplasia and hypoplasia were detected in 30% and 24% of the patients respectively. Dysplastic changes in bone marrow aspiration are illustrated in table 1.

**Table 1:** Dysplastic changes in bone marrow

**Bone marrow findings**		**Percentage**	**Total percentage**
One lineage Dysplasia	Megalodyserythropoiesis	25	31
	Dysmyelopoiesis	2	
	Dyserythropoiesis	4	
Two lineage Dysplasia	Dyserythromyelopoiesis	8	9
	Dysmegamyelopoiesis	1	
Three lineage Dysplasia	Dyserythromyelomegakaryopoiesis	10	10

Bone marrow biopsy examination of 67% of the patients was hypercellular, 19% had hypocellular and 13% had normocellular marrow. There were some degree of fibrosis in a few cases but only one of them had markedly fibrotic marrow that was diagnosed as MMM. 

By considering peripheral blood and bone marrow examination, diagnosis of patients were; MDS (33%), MDS Vs. MA (23%), leukemia (18%) (13 cases), hypoocellular marrow with no other specific finding (7%), aplastic anemia (3%), normocellular marrow with no specific finding (3%), Lymphoma (3%), leishmaniasis (3%), Hairy cell leukemia (1%), MDS/MPN (1%), MMM (1%) and MM (1%). The cellularity of the marrow in different diagnoses is depicted in table 2. The bone marrow cellularity in different types of MDS as detected in this study is shown in table 3.

**Table 2:** Bone marrow cellularity in different categories of patients diagnoses

**Diagnosis**	Cellularity^*^				**Summative percentage**
	**Hypocellular**	**Normocellular**	**Hypercellular**	**Fibrotic**	
Normocellular marrow	0	3	0	0	3
Acute leukemia	1	0	17	0	18
MDS	5	4	24	0	33
Aplastic anemia	6	0	0	0	6
MDS/MPN	0	0	1	0	1
Leishmaniasis	0	3	0	0	3
LPD-HCL	0	1	0	0	1
MMM	0	0	0	1	1
MM	0	0	1	0	1
LPD	0	0	3	0	3
hypocellular marrow	7	0	0	0	7
MDS VS MA	0	3	20	0	23
Total	19	13	67	1	100

*Numbers in percent

**Table 3:** The bone marrow cellularity in different types of MDS

**Cellularity**	**Type**				**Total** **Percentage**
	RARS^*^	RCMD**	RAEB***	**MDS evolving to acute leukemia**	
HypoCellular	0	3	2	0	5
NormoCellular	0	2	2	0	4
HyperCellular	1	14	8	1	24
Total Percentage	1	19	12	1	33

^*^Refractory anemia with ring sideroblasts (RARS), ^**^Refractory cytopenias with multilineage dysplasia (RCMD); ^***^Refractory anemia with excess of blasts (RAEB)

Cytogenetic study on the hundred patients showed 31% abnormal karyotype from which 41.9% (13 patients) were found in MDS patients and 35.4% (11 patients) in leukemic patients. The remainders were observed in various diseases containing lymphoma (6.45%), MMM (3.2%), MDS/MPN (3.2%), MDS versus MA (6.45%) and hypocellular group (3.2%). Due to the study plan, follow of the patients towards an understanding of their prognosis and relapse or progression of their disease were not carried out.

**Figure 1:**
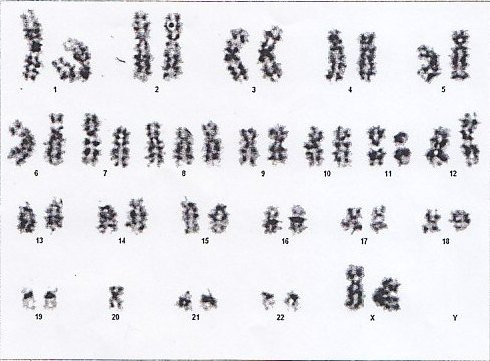
Complex karyotype: *46XX,+8,-20,der18,del(11)(p15),t(5;12)(q31;p13), t(7;12)(p36;p13)*

**Figure 2:**
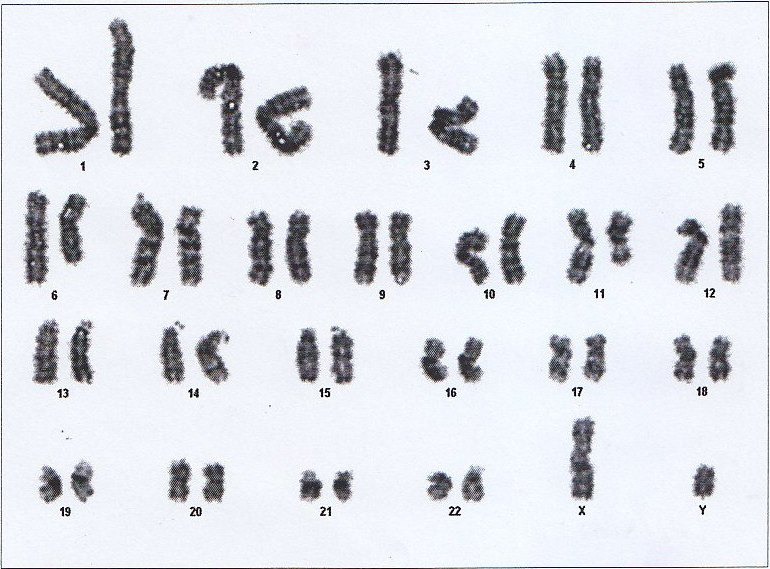
Complex karyotype: *46,XY,del(6)(q23), del(7)(q22), del(11)(q14)*

**Figure 3:**
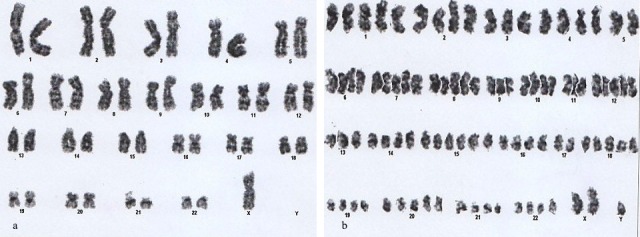
45,X,-Y[3]/46,XY[7]/Tetraploidy[5]: (a) Absence of Y , (b) Tetraploidy

**Figure 4:**
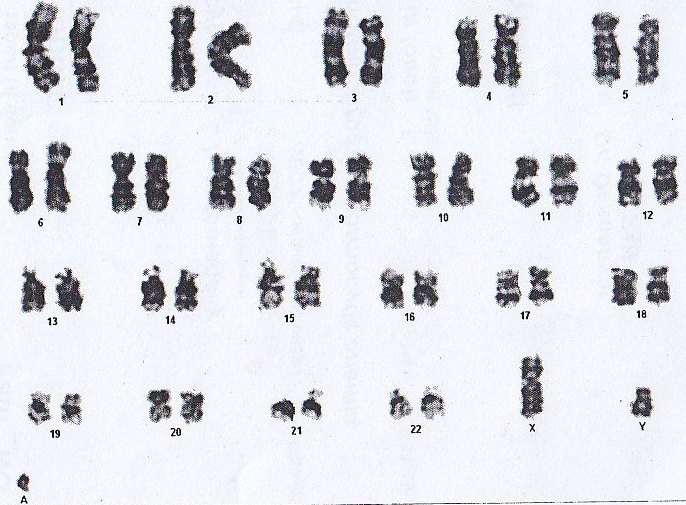
Karyotype of the patient with Marker chromosome : *47,XY,+mar[3]/46,XY[12]*

In table 4 the frequency of normal and abnormal karyotyping in patients is represented. In detail, the karyotype of patients in different diagnoses categories is illustrated in table 5. Figures 1 to 4 show the karyotype illustrations of some patients. Complex karyotypes are shown in figures 1 and 2. In figure 3, an interesting abnormal karyotype that illustrate s –Y in one clone and hyperploidy in another clone is shown.  A marker chromosome is revealed in figure 4. Detailed clarification of the cases is included in the “Discussion” section.

**Table 4:** Descriptive data of karyotype study findings in different categories of diagnoses

**Diagnosis**	**Karyotype**		**Total**
	**Normal**	**Abnormal**	
Normocellular	3	0	3
Leukemia	7	11	18
MDS	20	13	33
Aplastic	6	0	6
MDS/MPN	0	1	1
Leishmaniasis	3	0	3
HCL	1	0	1
MMM	0	1	1
MM	1	0	1
Lymphoma	1	2	3
Hypocellular marrow	6	1	7
MDS VS MA	21	2	23
Total	69	31	100

**Table 5:** Abnormal karyotype findings in different categories of diagnoses

**Karyotype finding**	**Diagnosis**	**Percentage**
44,XY,-4,-5,-21,+mar,del(7)(q32),del(18)(p11)[20]/56XXYY, +1, +5,+12,+13,+20,+22,del(7) )(q32), del(18)(p11)[5]	MDS, RCMD	1
46,XX,t(9;?15)(q31;q22)	MDS, RCMD	1
46,XY,dup(21)(q21)	MDS, RCMD	1
45,X,-Y	MDS, RCMD	1
93,XXXYY[4]/46,XY[12]	MDS, RCMD	1
46,XX,t(7;20)(p15;q13),del(7)(q22), dup(21)(q21)	MDS, RCMD	1
45,X,-Y[9]/46,XY[1]	MDS, RCMD	1
46,XY,16qh+	MDS, RCMD	1
46,XY,del(6)(q23), del(7)(q22), del(11)(q14)	MDS, RAEB	1
46,XY,+1,der(1),t(1;16)(p11;q11)	MDS, RAEB	1
46XX,t(5;20)(q15;q13)	MDS, RAEB	1
45,XX,-7	MDS, RAEB	1
46XX,+8,-20,der18,del(11)(p15),t(5;12)(q31;p13), t(7;12)(p36;p13)	MDS, RAEB	1
46,XY,t(15;17)(q22;q11)	APL(AML,M3)	4
46,XY ,del(1)(q23)	AML(non M3)	1
47,XY,+mar[5]/46,XY[10]	AML(non M3)	1
46,XY, t(2;13)(q31;p11), t(11;17)(q23;p25),der(12)	AML(non M3)	1
46,XX,t(3;5)(q25;p34), del(7)(q22),	AML(non M3)	1
48,XY,+21c,+22,del(9)(q23)	AML(non M3)	1
54,XX,+5,+6,+14,+20,+21,+22,+mar[2]/46,XX[13]	ALL(Precursor B cell)	1
46,XY,der(1),dup(1)(p32)	ALL(Precursor B cell)	1
45,X,-Y[3]/46,XY[7]/Tetraploidy[5]	MA Vs. MDS	1
46,XY,inv(9)(p13;q12)	MA Vs. MDS	1
46,XX,i(17)(q10;q10)[8]/46,XX[17]	Lymphoma(Diffuse large B cell)	1
47,XY,+21,der(14),add?(14)(q32)[6]/46,XY[10]	Lymphoma(Diffuse large B cell)	1
47,XY,+mar[3]/46,XY[12]	Hypocellular	1
47,XY,+mar[2]/46,XY[18]	MDS/MPN	1
46,XY,+4,-8, t(7;16)(q32;q13)[2]/Tetraploidy [5]/ 46,XY[17]	MMM	1

Amongst the 19 MDS patients with RCMD, 8 (42.1%) had abnormal karyotype. Five out of thirteen (38.5%) patients with REAB had karyotype abnormality. There was just one patient with RARS that had no karyotype abnormality. The data are shown in detail in table 5. Among the 18 patients with acute leukemia 13 (72%) were in AML group, where 9 (69%) of them had karyotype abnormality. The remaining leukemic patients (23%) had ALL from which two exhibited karyotype abnormality.

## Discussion

New-onset pancytopenia can be caused by a vast variety of etiologies. Clinical evaluation is the bases for diagnosis in many patients,^11^ however, bone marrow examination is recommended for detecting the cause in most patients. Although bone marrow examination often shows an underlying cause of pancytopenia, it is not always sufficient and conclusive. For a better understanding of various disorders that may cause pancytopenia, additional tests such as immunophenotyping, cytogenetic and molecular study may be necessary. In this study 100 patients with pancytopenia were studied. Age, gender wise incidence, some clinical findings, peripheral blood picture, bone marrow aspiration and biopsy findings, different causes of pancytopenia and the presence or absence of karyotype abnormality in various diagnoses were investigated in all the cases.

In the present study, the superior RBCs findings was normocytic normochromic anemia (48%) followed by macrocytic anemia (30%). However, in a study by Gayathri et al. dimorphic anemia (37.5%) followed by macrocytic anemia (31.7%) were the predominant peripheral blood picture.   

One of the most practical results of this study was the presence of blast in peripheral blood of 10% of the patients. 80% of these were diagnosed as acute leukemia and 20% were categorized in MDS with excess blast. It is important to note that the percentage of blasts in peripheral blood of these patients was below 10%, thus definite diagnosis was not feasible according to peripheral smear findings.

In previously performed investigations on etiology and prevalence of pancytopenia, the results are reported to be very different. In a study by Gayathri et al.,^3^ Megaloblastic anemia was the commonest cause of pancytopenia (70.04%) and aplastic anemia was the second common cause (17.26%). In a study by Santra et al.,^4^ AA was the most frequent cause (22.72%). In a study by Jalaee khoo et al. in Iran,^12^ the MDS after acute leukemia, AA, MA was the forth common cause of 188 adult patients with pancytopenia (9.5%) . In contrast, the distribution of diagnosis in this study was entirely different, namely; MDS (33%), MDS v/s MA (23%), Leukemia (18%), Hypocellular marrow (7%) and Aplastic anemia (6%). Such variation in data may be in part due to the different source of patient selection.

Pancytopenic patients, depending on the underlying cause, can have hyperplasic, hypoplastic or normocellular marrow. In the present study, the majority of patients (67%) had hypercellular marrow, 19% hypocellular, 13% normocellular and only one patient had completely fibrotic marrow. In a study by Santra et al.^4^ on 111 adult patients (13-65 y) done in India, hypocellular marrow was the major picture (45.95%) followed by hypercellularity and normocellularity with 37.83% and 16.22% respectively. However, similar to the present study, in Gayathri study^3^ hypercellularity was the most frequent bone marrow findings.

In MDS category, 84.9% of patients had hypercellular or normocellular marrow whereas hypocellularity contained 15.1% of the cases. This finding is compatible with previous studies.^13^

The other various etiologies that was identified in this study were; lymphomatous involvement (3%), kala azar (3%), multiple myeloma (1%), myeloid metaplasia with myelofibrosis (MMM) (1%), MDS/MPN (1%) and Hairy cell leukemia (1%). 3% of the cases in this study had normocellular marrow without any abnormal bone marrow findings. These patients were designated as normocellular marrow without definite diagnosis. In a study by Pathak et al. in Nepal on 102 pancytopenic patients, almost the same result was obtained as bone marrow examination in 4.9% was normocellular with no diagnosis.^7^

In some patients even with the use of all these methods, a definite diagnosis could not be reached. One group (7%) of these patients was designated as hypocellular marrow because of diluted aspiration and hypocellularity of the marrow. Also, occasionally due to the presence of mild degree of dysplasia, differentiation between hypocellular MDS versus aplastic anemia or other rare causes of hypocellularity was not possible. In another group (23%), with hypercellular or normocellular marrow and only megaloblastic or mild megalodyserythropoietic change without any other lineage dysplasia, it was not possible to separate MDS from megaloblastic anemia (MA) with certainty and cytogenetic finding except in two of them was normal. Therefore, these patients were grouped as MDS versus MA.

The most important findings of this study were bone marrow and cytogenetic findings. In this regard, no literature could be found with similar purpose that considered karyotype findings in pancytopenic patients. Cytogenetic study on the 100 patients showed 31% abnormal karyotype from which 41.9% (13 patients) were found in MDS patients and 35.4% (11 patients) in leukemic patients. The remainder was seen in various diseases containing lymphoma (6.45%), MMM (3.2%), MDS/MPN (3.2%), MDS versus MA (6.45%) and hypocellular group (3.2%). 

In MDS patients (that by criteria were primary MDS not secondary), the abnormal karyotype was present in 13 patients out of 33 (39.4%). In a study by Pozdnyakova et al. abnormal karyotype was detected in 45% of MDS patients.^14^ In another study by Vundinti et al. 54.48% of patients had abnormal karyotypes.^15^

Complex karyotype, that defines at least three simultaneous karyotype abnormalities in one cell clone independent to each other,^16^ were seen in 30.8% of current MDS patients being the most frequent karyotype abnormality of them. The results of this study is in agreement with the results of the study done by Haase^16^ which shows complex karyotype in 30% of abnormal karyotypes of MDS patients. In a study by Pozdnyakova et al. the percentage of complex karyotype has been increased to 39.5%.^14^ The other most frequent chromosomal abnormalities of MDS in a study by Haase was -7/7 q- with 25% frequency of abnormal karyotypes in isolated ones, with one additional karyotype or in complex karyotypes. In the current study -7/7 q- was present in 30.8% of abnormal karyotypes (alone and in complex kartoyotype). Other abnormal karyotypes that were mentioned in Haase’s study including +8, -Y, -5, -17/17 q-, +1, -21 and -5 q were also detected in this study.

Interestingly, two novel karyotype abnormalities were found in 2 of the 13 MDS patients with abnormal karyotype including t(5;20)(q15; q13) and t(9; ?15)(q31; q22).

From 23 patients in MDS versus MA, two patients had abnormal karyotypes in which one of them (92 years old) showed –Y in one clone and hyperploidy in another clone. This patient was thus diagnosed as MDS. Although loss of Y chromosome is one of the frequent cytogenetic findings singly or in complex karyotypes, but it is also an age related process that can be seen normally in old patients.^14^^,^^16^^,^^17^

From 7 patients with Hypocellular marrow, only one had abnormal karyotype finding as marker chromosome.  While this finding is not a diagnostic for MDS, but it is worth mentioning that such chromosome abnormality gives indicative confidence for extra follow up of patients.

Two patients with normocellular marrow without any dysplasia, abnormal findings and definite diagnosis had normal karyotype. Patients with diagnosis of leishmaniasis also had expectedly normal karyotype. 

Even though the patients were not followed up towards obtaining their eventuality and treatment implication, it is worth noting that in MDS and MDS/MPN for predicting survival and risk of acute leukemic transformation percentage of blasts, cytogenetics and more than one cytopenia are used. Generally, blast count >5% raises threat and >10% makes more risk; complex karyotype abnormalities or chromosome 7 abnormalities causes high risk; low risk observations are: del(5q), isolated del(2q), -Y, and normal cytogenetics ; other cytogenetic findings are intermediate risk. Also in AML, cytogenetic study is useful for diagnosis, classification and treatment purposes especially to signify the t(15;17) that has different treatment from other AML subtypes.  In ALL, cytogenetic abnormalities can have an effect on prognosis and response to treatment as hyperdioploidy has a better outcome than hypodiploidy.^18^

## Conclusion

The present investigation concludes that cytogenetic study on bone marrow aspiration can be useful in diagnosis of few patients with vague bone marrow finding. However, there are also cases where diagnosis even with implementing bone marrow examination and cytogenetic analysis is not possible. Such patients require more clinical follow up and investigation. Finally, additional studies with larger sample size and prolonged follow up are essential towards obtaining a comprehensive data and a better perspective. 
